# Stabilization of Port Hamiltonian Chaotic Systems with Hidden Attractors by Adaptive Terminal Sliding Mode Control

**DOI:** 10.3390/e22010122

**Published:** 2020-01-19

**Authors:** Ahmad Taher Azar, Fernando E. Serrano

**Affiliations:** 1 Robotics and Internet-of-Things Lab (RIOTU), Prince Sultan University, Riyadh 12435, Saudi Arabia; 2 Faculty of Computers and Artificial Intelligence, Benha University, Benha 13511, Egypt; 3 Universidad Tecnologica Centroamericana (UNITEC), Tegucigalpa 11101, Honduras; feserrano@unitec.edu or

**Keywords:** chaos, hidden attractors, port hamiltonian systems, adaptive control, adaptive terminal sliding mode control

## Abstract

In this study, the design of an adaptive terminal sliding mode controller for the stabilization of port Hamiltonian chaotic systems with hidden attractors is proposed. This study begins with the design methodology of a chaotic oscillator with a hidden attractor implementing the topological framework for its respective design. With this technique it is possible to design a 2-D chaotic oscillator, which is then converted into port-Hamiltonia to track and analyze these models for the stabilization of the hidden chaotic attractors created by this analysis. Adaptive terminal sliding mode controllers (ATSMC) are built when a Hamiltonian system has a chaotic behavior and a hidden attractor is detected. A Lyapunov approach is used to formulate the adaptive device controller by creating a control law and the adaptive law, which are used online to make the system states stable while at the same time suppressing its chaotic behavior. The empirical tests obtaining the discussion and conclusions of this thesis should verify the theoretical findings.

## 1. Introduction

Chaos theory represents a new field of research based on qualitative and empirical analysis of chaotic a periodic behavior [1,2,3,4,5,6,7]. A dynamic system is called chaotic when it fulfills the three characteristics of constraints, constant repetitions, and responsive dependent conditions.

Chaotic systems with hidden attractors have been extensively studied during recent years because the vast amount of physical systems in which this phenomenon is found [8,9,10,11]. Self-excited attractors are those kind in which the domain of attraction is at least one of the equilibrium points, whereas hidden attractors are those whereas the domain of attraction is not balanced [12]. In the literature there are many studies in which hidden attractors are analyzed, for example, in [13], a new 3-D chaotic system with hidden attractor is designed and analyzed in which the eigenvalues and Lyapunov exponents are obtained for dynamical analysis purposes. Other examples can be found in [14], in which a simple 4-D chaotic system is evinced in the presence of a hyperbolic cosine nonlinearity, where some phenomenons such as multistability, antimonotonicity, and quasi-periodic orbits are analyzed. In [15], the multistability phenomenon is related to the occurrence of unpredictable attractors which are called hidden attractor. In that study a deep analysis of this kind of complex chaotic systems is offered showing the fundamentals of chaotic systems with hidden attractors. In [16], a novel hidden chaotic attractor is shown in which a new category of multi stable systems is analyzed. This category is a system with extreme multi-stability, so the authors showed a new five-dimensional chaotic system with a hidden attractor. In [17], another interesting study is found in which hidden attractors, system with no equilibria and multi-stability are analyzed. In [18], important results are shown in which a fractional order multi-stable four dimensional system with hidden attractor is shown considering that hidden attractor are even found in fractional order chaotic systems and not only in integer order ones. Hidden attractors found in circuits are of great importance not only because of the mathematical context, but also in view of their representation. For example in [19], the multi-stability and hidden attractors in relay systems with hysteresis are studied. In that study it is shown how the equilibrium point in a relay system disappear in a boundary equilibrium bifurcation. In [20], another interesting example of a fractional order chaotic system with hidden attractors is presented. The control, circuit realization and fractional order analysis of hidden attractors in a self-excited homopolar disk dynamo is shown. Then, in [21], the dynamic analysis and circuit implementation of a fractional order chaotic system is shown.

One of the main contribution of this study is the design and stabilization of chaotic port Hamiltonian systems with hidden attractors. Related reference to chaotic port Hamiltonian systems can be found in [22], where a new class of conservative chaotic system with multi-stability is designed, where the dynamic properties of this new system are analyzed by its equilibrium points and Lyapunov dimensions among others. Another interesting example can be found in [23], where another novel conservative port Hamiltonian chaotic system in which the route from periodic to quasi-periodic chaos and strong chaos is shown. Finally, in [24], a 3-D Hamiltonian chaotic system is shown considering conservative and dissipative chaotic flows.

In this paper, we intend to design an adaptive terminal sliding mode controller for the studied system. For example, in [25,26,27,28], different adaptive terminal sliding mode controller approaches have been implemented for different kinds of system, specifically, unmanned aerial vehicles and robotic systems. Note that no study has been reported for the stabilization of port Hamiltonian chaotic systems, but these strategies are useful for the present study. Finally, in [29,30], adaptive fast terminal sliding mode controller strategies are implemented in the control of robotic manipulators and unmanned aerial vehicles, respectively.

In this study, the design and stabilization of port Hamiltonian chaotic systems with hidden attractor is shown. Next, design methodologies for the Hamiltonian chaotic port [31] are implemented where the design of chaotic systems with hidden attractors of all dimensions can be obtained by designing a suitable Hilbert space, with its topology. The adaptive terminal sliding mode controller proposed in this paper is designed by establishing an appropriate sliding manifold to find the control law and then the adaptive control laws are obtained by selecting an appropriate Lyapunov function. The designed controller is used to balance a 2-D port Hamiltonian chaotic system, which was constructed according to the method shown in this paper and then a 4-D chaotic system with a hidden attractor as shown in [12].

This paper is organized as follows. Section 2 describes the design of chaotic oscillator system with hidden attractor and problem formulation. In Section 3, Adaptive Terminal Sliding Mode Controller Design is proposed. Section 4 details the numerical experiments illustrating the main results derived in this research paper with comparative analysis. In Section 5, discussion of the results is presented. Section 6 contains the main conclusions of this work with future directions.

## 2. Chaotic Oscillator System with Hidden Attractor Design and Problem Formulation

In this section, the chaotic oscillator with attractor design is shown along with the problem formulation. First, a 2-D chaotic system with hidden attractor is designed and transformed to a port Hamiltonian form along with a 4-D chaotic system. The port Hamiltonian representation is shown considering the position and momentum. Then, in the next section, the stabilization of a chaotic system in port Hamiltonian form is presented.

### 2.1. Chaotic Oscillator System with Hidden Attractor Design

For the design of a chaotic system with hidden attractors, the following Hilbert space are needed. Consider the following Hilbert space with inner product defined as <x,y>

(1) $$\begin{array}{ccc} H & = & \left[h_{1},h_{2},\ldots ,h_{p}\right]\\ G & = & \left[g_{1},g_{2},\ldots ,g_{q}\right] \end{array}$$

where $h_{i}\in R^{n}$ and $g_{i}\in R^{n}$. Now define the following set as

(2) $$M=\{k_{i}\in H\times G\;:\;<k_{1},k_{2}>/<k_{1}\left(0\right),k_{2}\left(0\right)>\to \infty \}$$

where *i* = 1,2 and *M* define the chaotic system solution pairs to allow the dynamic system to join the chaotic system in light of the initial conditions [32]. The area of attraction for the chaotic hidden system must have the dynamic system solution outside the border of the balance points as described by the following set [33],

(3) $$D_{0}=\left\{x_{0}\in D\;:\;\mathrm{if}\;x\left(0\right)=x_{0}\;\mathrm{then}\;lim_{t\to \infty }x\left(t\right)=a\right\}$$

where *a* is a point outside the boundary of the equilibrium points. Now selecting

(4) $$f_{ir}\subset M$$

Then,

(5) $$F_{r}=\overset{N}{\underset{i=1}{⋂}}f_{ir}$$

for r=1…n; *N* is a specific number of subsets selected to find a domain of attraction outside of the boundary of the equilibrium points so as to find the set of attraction for the designed oscillator. Therefore, for the chaotic oscillator defined by the following dynamic system,

(6) $$\frac{dx}{dt}=F\left(x,t\right)$$

From (5), the elements of F(x,t) are found by implementing

(7) $$F\left(x,t\right)=\left[\begin{array}{c} \nabla .F_{1}\left(x\right)\\ \nabla .F_{2}\left(x\right)\\ \vdots \\ \nabla .F_{n}\left(x\right) \end{array}\right]$$

where $x={\left[x_{1},x_{2},\ldots ,x_{n}\right]}^{T}$ and the divergence is defined as

(8) $$\nabla .F_{r}\left(x\right)=\frac{\partial F_{r1}}{\partial x_{1}}+\frac{\partial F_{r2}}{\partial x_{2}}+\ldots +\frac{\partial F_{rn}}{\partial x_{n}}$$

for r=1,…,n. With these definition chaotic oscillators with hidden attractors of any dimension can be designed.

#### 2.1.1. 2-D Chaotic Oscillator with Hidden Attractor Design

The following vector fields were obtained by (5)

(9) $$\begin{array}{ccc} F_{1} & = & \left[\begin{array}{c} x_{1}x_{2}\\ x_{1}^{3} \end{array}\right]\\ F_{2} & = & \left[\begin{array}{c} x_{1}x_{2}sin\left(x_{2}\right)\\ x_{1}x_{2}sin\left(x_{2}\right) \end{array}\right] \end{array}$$

by obtaining the divergences of (9), the following results are obtained,

(10) $$\begin{array}{ccc} \nabla .F_{1}\left(x\right) & = & \frac{\partial F_{11}}{\partial x_{1}}+\frac{\partial F_{12}}{\partial x_{2}}=x_{2}\\ \nabla .F_{2}\left(x\right) & = & \frac{\partial F_{21}}{\partial x_{1}}+\frac{\partial F_{22}}{\partial x_{2}}=x_{2}sin\left(x_{2}\right)+x_{1}sin\left(x_{2}\right)+x_{1}x_{2}cos\left(x_{2}\right) \end{array}$$

Therefore, the following dynamical system is obtained and later will be represented in port Hamiltonian form:

(11) $$\frac{dx}{dt}=\left[\begin{array}{c} ax_{2}\\ bx_{2}sin\left(x_{2}\right)+cx_{1}sin\left(x_{2}\right)+dx_{1}x_{2}cos\left(x_{2}\right) \end{array}\right]$$

where the constants a,b,c,d are selected according to the system initial conditions as explained in (2) and (3). The constants values are $a=-30\times 10^{-3}$, $b=40\times 10^{-1}$, $c=-9\times 10^{-5}$, and $d=-9\times 10^{-10}$.

#### 2.1.2. 4D-Chaotic System with Hidden Attractor

The second studied chaotic system is found in [12]. Consider the following state variable $x={\left[x_{1},x_{2},x_{3},x_{4}\right]}^{T}$ so this system is represented as

(12) $$\frac{dx}{dt}=\left[\begin{array}{c} x_{2}\\ x_{3}\\ x_{3}+ax_{2}x_{4}-x_{3}x_{4}\\ x_{1}x_{2}+bx_{2}x_{3} \end{array}\right]$$

where the constants are selected as a=−1 and b=1. Similar to the previous chaotic system, this system will be represented later in port Hamiltonian form.

### 2.2. Problem Formulation

**Figure 1:**
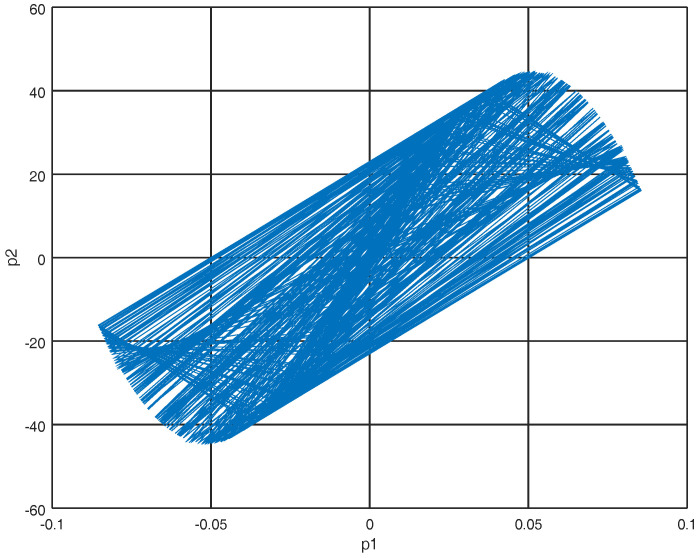
Phase portrait of $p_{1}$ and $p_{2}$.

**Figure 2:**
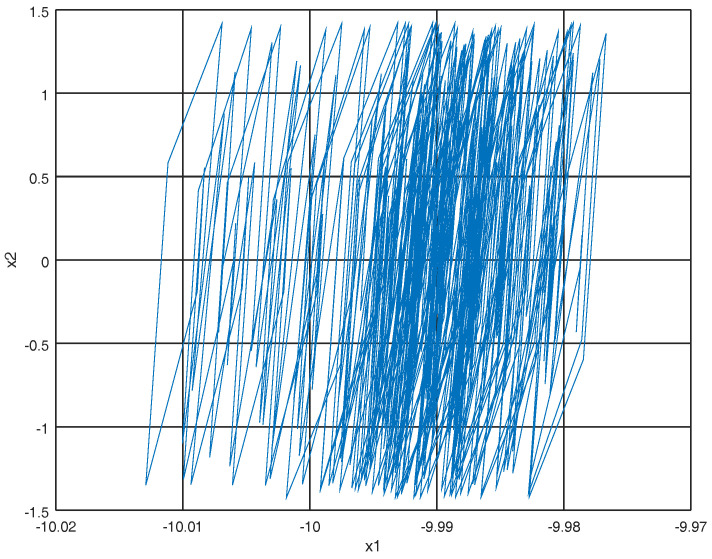
Phase portrait of $x_{1}=q_{1}$ and $x_{2}=q_{2}$.

Consider the following port-Hamiltonian formulation only with a conservative part with q=x [22,23,24,31]:

(13) $$\dot{X}\left(t\right)=J\left(X\left(t\right)\right)\left[\frac{\partial H(X(t))}{\partial X(t)}\right]+G\left(X\left(t\right)\right)u\left(t\right)$$

where $X\left(t\right)={\left[q^{T},p^{T}\right]}^{T}$, $X\left(t\right)\in R^{m}$, $u\left(t\right)\in R^{o}$, $J\left(X\left(t\right)\right)=-J\left(X\left(t\right)\right)\in R^{m\times m}$ is the conservative matrix and $G\left(X\left(t\right)\right)\in R^{m\times o}$, which is selected as an identity matrix of appropriate dimension. The matrix J(X(t)) is given as [31]

(14) $$J\left(X\left(t\right)\right)=\left[\begin{array}{cc} 0 & I\\ -I & 0 \end{array}\right]$$

with identity and zero matrices of appropriate dimension. The Hamiltonian and Lagrangian are given by [31]

(15) $$\begin{array}{c} H\left(X\left(t\right)\right)=\dot{q}{\left(t\right)}^{T}p\left(t\right)-L\left(X\left(t\right)\right)\\ L\left(X\left(t\right)\right)=K\left(\dot{X}\left(t\right)\right)-U\left(X\left(t\right)\right) \end{array}$$

where U(X(t)) is the potential energy. Note that $\frac{\partial U(X(t))}{\partial X(t)}=F\left(X\left(t\right)\right)$ so the momentum $\dot{p}=M\ddot{q}$ where *M* is an appropriate mass matrix. Representing system (11) in the port Hamiltonian representation (13) with initial conditions $X_{0}={\left[-10,1,0,0\right]}^{T}$, the phase portraits can be viewed in Figure 1 and Figure 2.

**Figure 3:**
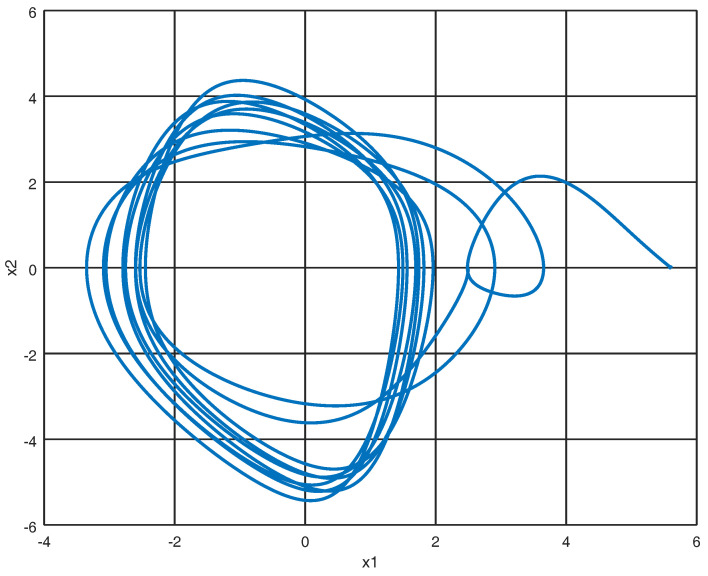
Phase portrait of $x_{1}=q_{1}$ and $x_{2}=q_{2}$.

**Figure 4:**
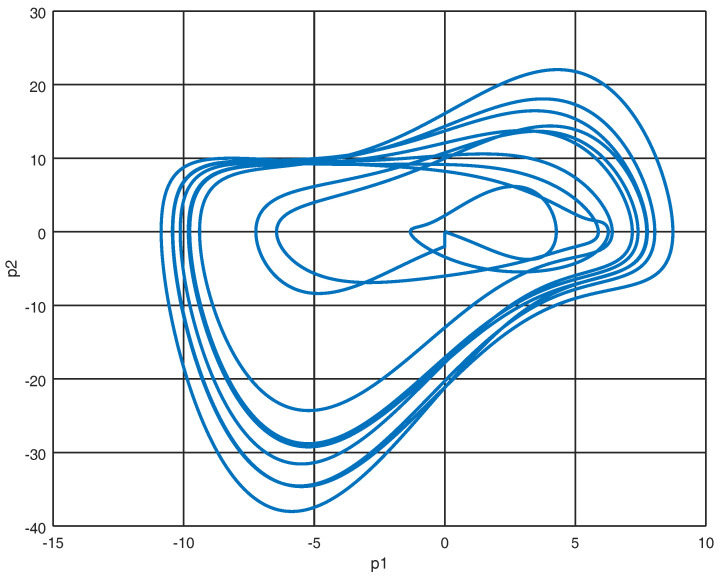
Phase portrait of $p_{1}$ and $p_{2}$.

The system (12) represented in port Hamiltonian form as shown in (13) with initial conditions $X_{0}={\left[2.5,0,-1,-0.4,0,0,0,0\right]}^{T}$ yields the phase portraits as shown in Figure 3 and Figure 4.

## 3. Adaptive Terminal Sliding Mode Controller Design

The designed adaptive terminal sliding mode controller (ATSMC) eliminates the chaotic behaviour even in the presence of hidden attractors. This controller stabilizes the system in the equilibrium points while eliminating the chaotic oscillation. This ATSMC is based on the discovery of an acceptable control law and a suitable adaptive law [25,27,28,29]. Consider the following variable and its derivative from (13) with G(X(t))=I:

(16) $$\begin{array}{ccc} s(t) & = & X(t)\\ \dot{s}\left(t\right) & = & \dot{X}\left(t\right)=J\left(X\left(t\right)\right)\left[\frac{\partial H(X(t))}{\partial X(t)}\right]+u\left(t\right) \end{array}$$

and the following sliding variable,

(17) $$\sigma \left(t\right)=k_{1}\dot{s}\left(t\right)+k_{2}s\left(t\right)+k_{2}sign\left(s\left(t\right)\right){∥s\left(t\right)∥}^{\rho }+k_{2}\lambda \left(s\left(t\right)\right)$$

where $k_{1},k_{2}\in R$, ρ is a constant. Therefore, consider the following [25].

(18) $$\lambda \left(x_{i}\right)=\left\{\begin{array}{ccc} x_{i}^{\frac{p_{r}}{q_{r}}} & if & {\hat{s}}_{i}=0\;\mathrm{or}\;{\hat{s}}_{i}\neq 0,\;\left|x_{i}\right|\geq \mu \\ t_{1}x_{i}+t_{2}sign\left(x_{i}\right) & if & {\hat{s}}_{i}\neq 0,\left|x_{i}\right|<\mu \end{array}\right.$$

All the constants of (18) are explained in [25,28,29]. The σ variable must be zero to ensure a sliding variable reaches its origin in the final time (reaching phase), while preserving its origin (sliding phase).

(19) $$k_{1}\dot{s}\left(t\right)+k_{2}s\left(t\right)+k_{2}sign\left(s\left(t\right)\right){∥s\left(t\right)∥}^{\rho }+k_{2}\lambda \left(s\left(t\right)\right)=0$$

Therefore, (19) becomes in

(20) $$k_{1}J\left(X\left(t\right)\right)\left[\frac{\partial H(X(t))}{\partial X(t)}\right]+k_{1}u\left(t\right)+k_{2}s\left(t\right)+k_{2}sign\left(s\left(t\right)\right){∥s\left(t\right)∥}^{\rho }+k_{2}\lambda \left(s\left(t\right)\right)=0$$

Therefore, the control law u(t) is

(21) $$u\left(t\right)=-J\left(X\left(t\right)\right)\left[\frac{\partial H(X(t))}{\partial X(t)}\right]-k_{1}^{-1}k_{2}s\left(t\right)+2k_{1}^{-1}k_{2}\lambda \left(s\left(t\right)\right)$$

and substituting (21) in (17) yields

(22) $$\sigma \left(t\right)=k_{2}sign\left(s\left(t\right)\right){∥s\left(t\right)∥}^{\rho }-k_{2}\lambda \left(s\left(t\right)\right)$$

With the previous result, the adaptive gain $k_{2}$ is obtained.

**Theorem** **1.** *The closed loop system which involves (13) is stabilized with the control law (21) and the adaptive gain ${\dot{k}}_{2}=-\dot{\sigma }{\left(t\right)}^{T}sign\left(s\left(t\right)\right){∥s\left(t\right)∥}^{\rho }+\dot{\sigma }{\left(t\right)}^{T}\lambda \left(s\left(t\right)\right)$.*

**Proof.**  Consider the following Lyapunov functional,

(23) $$V\left(\sigma ,k_{2}\right)=\frac{1}{2}\sigma {\left(t\right)}^{T}\sigma \left(t\right)+\frac{1}{2}k_{2}^{2}$$

and by taking the derivative of (23) yields

(24) $$\dot{V}\left(\sigma ,k_{2}\right)=\dot{\sigma }{\left(t\right)}^{T}\sigma \left(t\right)+k_{2}{\dot{k}}_{2}$$

Now substituting (22) into (24), the following result is obtained,

(25) $$\dot{V}\left(\sigma ,k_{2}\right)=\dot{\sigma }{\left(t\right)}^{T}k_{2}sign\left(s\left(t\right)\right){∥s\left(t\right)∥}^{\rho }-\dot{\sigma }{\left(t\right)}^{T}k_{2}\lambda \left(s\left(t\right)\right)+k_{2}{\dot{k}}_{2}$$

Therefore, by substituting the following adaptive law into (25) yields

(26) $${\dot{k}}_{2}=-\dot{\sigma }{\left(t\right)}^{T}sign\left(s\left(t\right)\right){∥s\left(t\right)∥}^{\rho }+\dot{\sigma }{\left(t\right)}^{T}\lambda \left(s\left(t\right)\right)$$

Thus, the derivative of the Lyapunov function (25) becomes

(27) $$\dot{V}\left(\sigma ,k_{2}\right)\leq 0$$

The closed loop stability of the system is ensured to drive the system’s variables to the equilibrium point and the sliding surface to the origin. □

The results obtained in the previous theorem show that the gain of $k_{1}$ is applied in the control law to make the sliding surface reach its origin in time. The only gain that can be tuned is $k_{2}$ because it is connected to the control law switching component.

## 4. Numerical Experiments

In this section, two numerical examples are shown. The first consists of the stabilization of the 2-D chaotic system (11) in port Hamiltonian form, and the second consists of the stabilization of a 4-D system (12) [12] represented in port Hamiltonian form. In the two experiment the proposed controller strategy is compared with the strategies shown in [25,29].

### 4.1. Experiment 1

**Figure 5:**
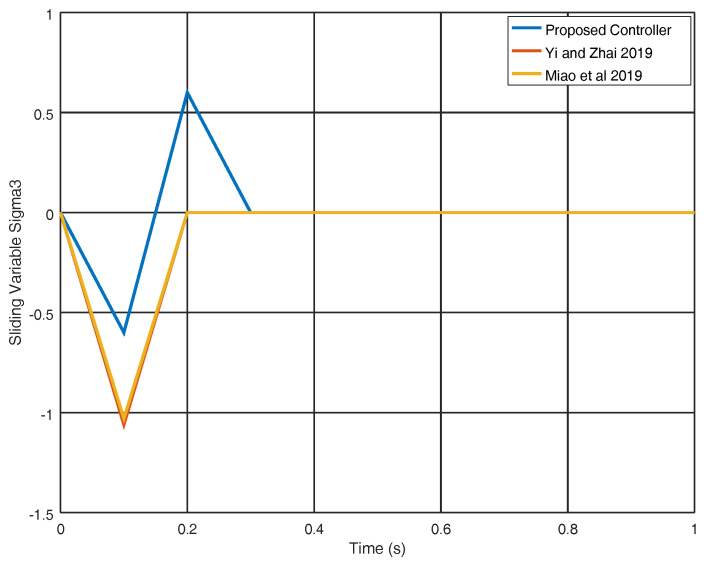
Sliding surface $\sigma_{3}$ fo the experiment 1.

**Figure 6:**
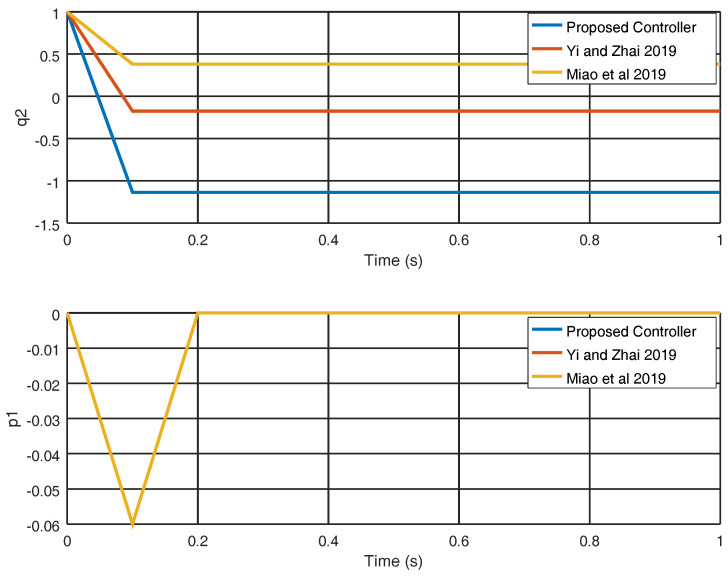
Evolution in time of the variables $q_{2}$ and $p_{1}$.

Figure 5 illustrates how the sliding variable $\sigma_{3}$ evolves and as can be seen; this variable originates without oscillations and is smaller than with the results achieved in [25,29]. In Figure 6, the variables $q_{2}$ and $p_{1}$ are evinced, and as can be noticed the equilibrium point is reached faster with the proposed strategy in comparison with the strategies shown in [25,29]. The results show that in contrast with the other methods, the variables correctly achieve the target final value.

**Figure 7:**
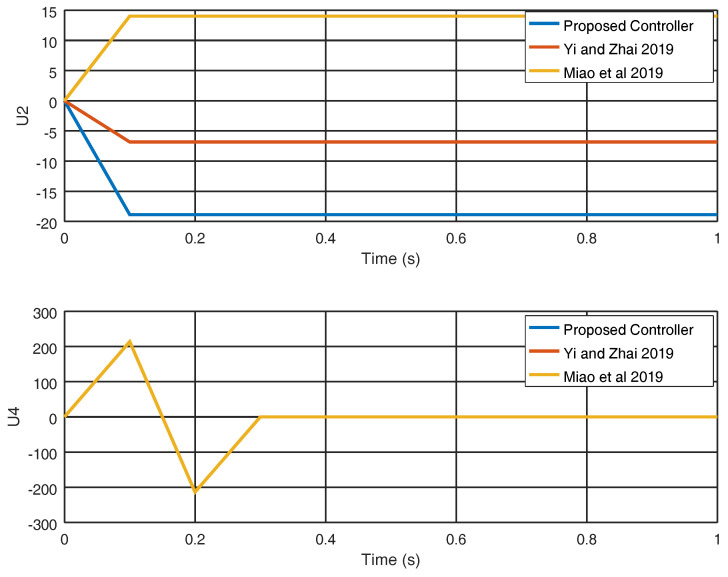
Input variables $u_{2}$ and $u_{4}$.

**Figure 8:**
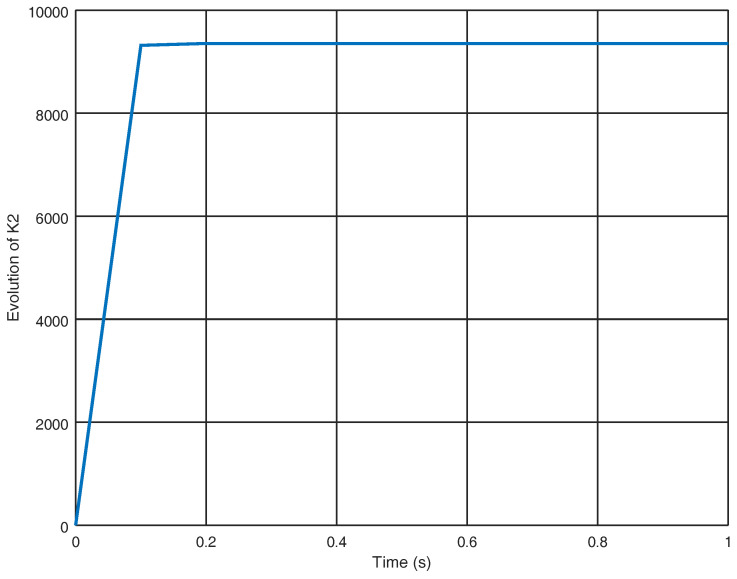
Evolution in time of the gain variable $k_{2}$.

The control inputs $u_{2}$ and $u_{4}$ are shown in Figure 7 and the control effort derived from the proposed controller is smaller in both variables than the control effort derived from [25,29]. Finally, in Figure 8, the evolution in time of the gain variable $k_{2}$ is shown and it is noticed that a significant final gain value is obtained in order to eliminate chattering and yield a smaller control effort.

### 4.2. Experiment 2

**Figure 9:**
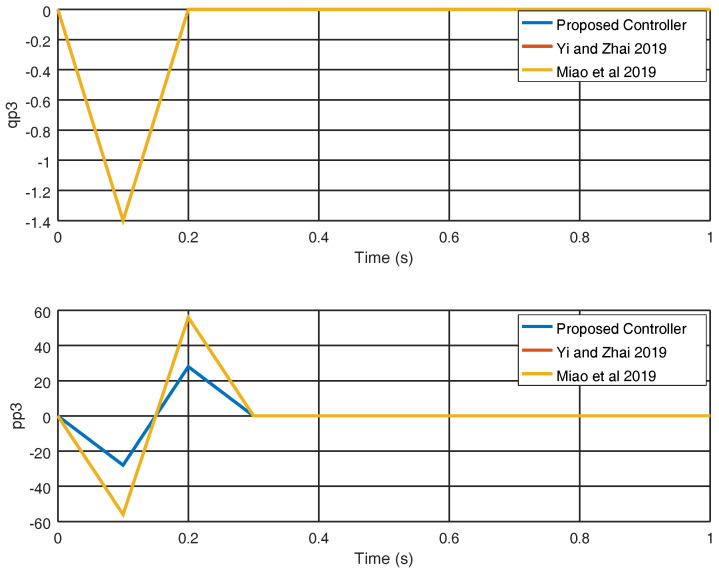
Evolution in time of the variables ${\dot{q}}_{3}$ and ${\dot{p}}_{3}$.

**Figure 10:**
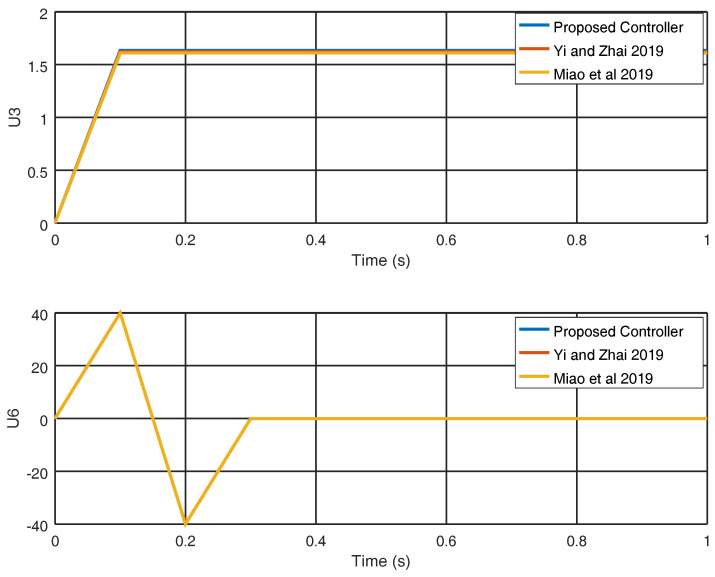
Evolution in time of the input variables $u_{3}$ and $u_{6}$.

In Figure 9, the evolution in time of the variables ${\dot{q}}_{3}$ and ${\dot{p}}_{3}$ is shown and a smaller overshoot is obtained with the proposed control strategy in comparison with the strategies shown in [25,29], proving the effectiveness of the proposed approach. In Figure 10, the input variables $u_{3}$, $u_{6}$ and the control effort obtained with the proposed control strategy are shown along with the results obtained in [25,29].

**Figure 11:**
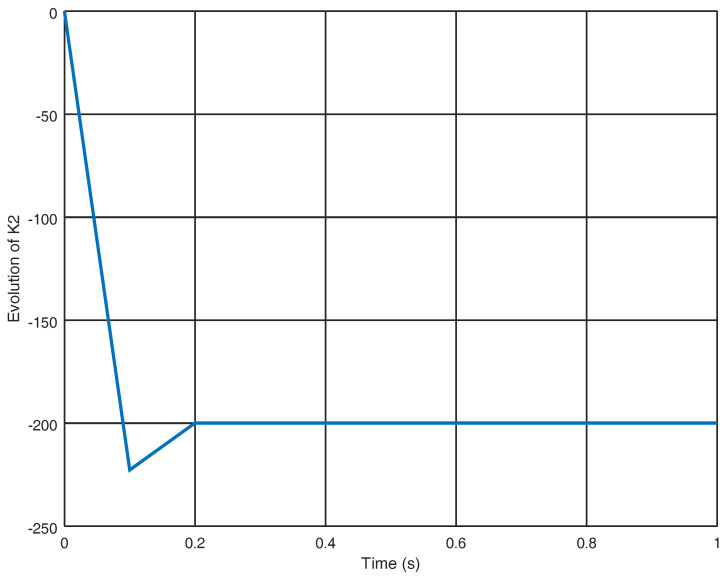
Evolution in time of the gain variable $k_{2}$.

**Figure 12:**
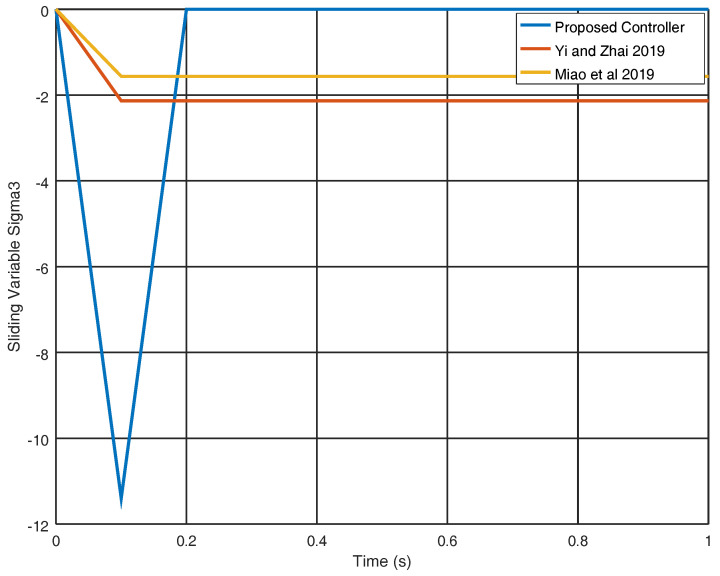
Evolution in time of the variable $\sigma_{3}$.

In Figure 11, the evolution in time of the gain variable $k_{2}$ is shown obtaining an adequate final value in order to avoid chattering with a reduced control effort. Finally, in Figure 12, the sliding variable $\sigma_{3}$ is shown in which it is proved that the sliding variable obtained by the proposed controller reaches the origin faster in contrast with the sliding variables obtained with the strategies shown in [25,29] which in this case do not reach the origin.

## 5. Discussion

This research, as concluded, has the aim of designing a new management strategy for chaotic port Hamilton system with hidden attractors. Considering that chaotic hidden attractors are a topic that has been intensively studied in recent years, it is important to develop new design methods and to perform dynamical analysis of these kinds of systems. In this paper, a novel design methodology to obtain chaotic systems with hidden attractors is proved successfully. This system is later transformed to a port Hamiltonian system which consider its energy properties. Besides, a chaotic system with hidden attractor [12] was used in this study for stabilization purposes, but before being transformed to a port Hamiltonian form. The results show that the system is stabilized better with our proposed control strategy in comparison with other strategies found in the literature. The convergence to the origin of the sliding variable is ensured by the proposed controller strategy. It must be noted that the strategies shown in [25,29] only lead the sliding variable in one of the experiments to the origin in finite time, whereas the proposed strategy shown in the study leads the sliding variable to the origin in both cases. This could occur by several reasons, for example by its equilibrium points and other dynamical properties of the port Hamiltonian chaotic system. Faster response, small control effort and fast convergence (reaching phase) prove the superior performance of the proposed control strategy.

## 6. Conclusions

In this paper, the design of an adaptive terminal sliding mode controller for chaotic port Hamiltonian systems with hidden attractor is proposed. A successful design methodology to obtain chaotic oscillator with hidden attractors is proposed first. This design methodology consists of the design of appropriate Hilbert spaces along with the topological framework to obtain the vector fields that are part of a nonlinear chaotic systems with hidden attractor. This technique allows to design and later convert a nonlinear chaotic 2D oscillator into a Port Hamilton system with a conservative part, taking into consideration the energy properties of the system as such property allows the proposed strategy to be successful, even if it is not based on strategies such as passivity-based control. As a second example, a 4-D chaotic system with hidden attractor [12] is analyzed and converted to a port Hamiltonian form. The proposed controller consists of designing an appropriate sliding variable in order to obtain the suitable control law which allows the system to reach the equilibrium points in finite time. Then, in order to ensure the closed loop stability of the system, the adaptive gain is obtained by selecting a suitable Lyapunov function. As explained in this paper, only one gain variable is adapted because it is only necessary to tune the gain variable related to the switching law. The other gain is implemented in the equivalent control law, so it is not necessary to tune it. The proposed controller can be used for other process control applications as one of the future directions. Also, hybrid control algorithms can also be applied as another future direction for this study.
